# Screening Clinical Changes for the Diagnosis of Early Knee Osteoarthritis: A Cross-Sectional Observational Study

**DOI:** 10.3390/diagnostics12112631

**Published:** 2022-10-30

**Authors:** Ana Alabajos-Cea, Luz Herrero-Manley, Luis Suso-Martí, Núria Sempere-Rubio, Ferran Cuenca-Martínez, Vicente Muñoz-Alarcos, Juan Alonso Pérez-Barquero, Enrique Viosca-Herrero, Isabel Vázquez-Arce

**Affiliations:** 1 Servicio de Medicina Física y Rehabilitación, Hospital La Fe, 46026 Valencia, Spain; 2 Exercise Intervention for Health Research Group (EXINH-RG), Department of Physiotherapy, University of Valencia, 46010 Valencia, Spain; 3 UBIC, Department of Physiotherapy, Faculty of Physiotherapy, Universitat de Valencia, 46010 Valencia, Spain

**Keywords:** osteoarthritis, knee osteoarthritis, early osteoarthritis, musculoskeletal disorders, risk factors

## Abstract

**Background:** The main objective was to evaluate differences in the clinical, motor, or functional variables in patients with Early Osteoarthritis (EOA) and individuals at risk of developing osteoarthritis (OA). **Methods:** A cross-sectional study was performed. All the participants were divided into two groups: EOA patients and healthy subjects (HS) at risk of developing OA. The main outcomes were clinical tests, such as those of knee morphology, instability, or proprioception; motor and functional variables, such as knee strength, range of motion, walking speed, and the sit-to-stand test; pain and disability, assessed through the Western Ontario McMaster Universities Osteoarthritis Index (WOMAC) and Knee injury and Osteoarthritis Outcome Score (KOOS) scales; and knee alignment and leg length inequality, assessed via X-ray images. **Results:** A total of 97 participants were included (54 EOA and 43 HS). Patients with EOA showed a greater presence of knee pain (*p* < 0.01). In addition, more EOA patients showed instability both in the left (*p* < 0.01) and right legs (*p* < 0.05). Regarding the knee alignment variable, significant differences were found (*p* < 0.04), with more patients with EOA diagnosed as possessing a varus alignment. In addition, EOA patients showed lower knee strength, since statistically significant differences were found regarding flexion and extension strength in the left leg (Mean Difference (MD): 12.92; *p* = 0.03; *d* = −0.46 and MD:7.81; *p* = 0.04; *d* = −0.39). Differences were found for the sit-to-stand test scores, showing lower results for the EOA group (MD: −1.91; *p* < 0.01; *d* = 0.54). **Conclusions:** The results of this research show statistically significant differences between patients with EOA and HS at risk of developing OA with respect to pain, disability, instability, knee strength, and the sit-to-stand test. Our results suggest that the evaluation of clinical, motor, and functional features could contribute to an early management of knee OA.

## 1. Introduction

Osteoarthritis (OA) is a common joint disease that causes disability and a reduction in patients’ quality of life. It is a leading cause of chronic pain and health-care utilization [1]. OA is characterized by cartilage loss, subchondral bone changes, synovial inflammation, and meniscus degeneration [2]. Basic research approaches have allowed for the identification of pathophysiological factors that determine the existence of OA. However, the main part of this research is performed in the late stage of OA, and the pathological processes involved in the early stages of joint disease are not well understood [3].

Previous studies suggest that many of the structural findings grounded in X-ray evidence in persons with knee OA are late phenomena, which occur after a considerable progression of the pathology [4,5]. In this regard, it has been suggested that the limited efficacy of non-surgical treatments for OA may be due partly to their use at a late point in the evolution of the disease, when structural deterioration is often advanced [4]. For these reasons, there is great scientific and social interest in ‘early osteoarthritis’ (EOA), as it is believed that identifying patients affected by EOA in order to initiate early interventions and therapeutic approaches could prevent disease progression and severe structural changes in the joint associated with later stages of OA [6].

However, this has not been studied in depth, and there is no consensus concerning what changes may be associated with EOA, which prevents the identification of these patients at an early stage. Since it has been shown that imaging studies may not be the best alternative in this regard, it has been suggested that some modifiable factors such as physical features and motor or functional variables could aid the identification of patients with EOA [7,8]. Our hypothesis is that we will find clear clinical, motor, and functional alterations in patients with EOA compared to healthy subjects. Therefore, the main objective of this study was to evaluate which clinical, motor, or functional variables could be related to the appearance of EOA.

## 2. Materials and Methods

### 2.1. Study Design

A cross-sectional study with a non-probabilistic sample was performed. The design followed the international recommendations for Strengthening the Reporting of Observational Studies in Epidemiology (STROBE) [9]. All participants received an explanation of the study procedures, which were planned according to the ethical standards of the Declaration of Helsinki and approved by an Ethics Committee (CEIm La Fe 2017/0147). Written informed consent was obtained from all participants before their inclusion.

### 2.2. Participants

Subjects were recruited and followed at Hospital La Fe, Valencia, Spain, within the H2020 project OACTIVE. The design of the data collection protocol started in November 2017, and the recruitment and follow-up of participants started in July 2018 and lasted until February 2021. All participants were divided into two groups: EOA and healthy subjects (HS) at risk of developing OA.

The subjects recruited were evaluated by a group of three experienced physical medicine and rehabilitation clinicians, always performing the same roles in the evaluation. The inclusion criteria for EOA patients were based on Luyten’s proposal for EOA classification, as the criteria used found a specificity of 76.5% for detection of clinical progression, making them valid criteria for research use [10]. The criteria were as follows: (a) patient-based questionnaires: Knee Injury and Osteoarthritis Outcome score—2 out of the 4 KOOS subscales (Pain, Symptoms, Function, or Knee-related quality of life) are needed to score “positive” (≤85%); (b) patients should present joint-line tenderness or crepitus in the clinical examination; (c) X-rays—Kellgren and Lawrence (KL) grade 0–1 for standing and weight-bearing (at least 2 projections—PA fixed flexion and skyline for patellofemoral OA) [11]. For matched controls, criteria were (a) patient age greater than or equal to 40 years; (b) body mass index greater than or equal to 25; (c) Kellgren and Lawrence score of 0–1. Exclusion criteria were the same for both groups: (a) any cognitive disability that hindered viewing of the audio-visual material; (b) illiteracy; (c) comprehension or communication difficulties, (d) insufficient Spanish language comprehension to follow measurement instructions; (e) presence of any rheumatic, autoimmune, or infectious pathology.

### 2.3. Outcome Measures

#### 2.3.1. Descriptive, Demographic Data and Control Variables

In this section, we included general demographic information included in other large databases, and well-established conventional predictors, such as gender, age, educational level, and marital status [8,12]. Personal history of hand or hip OA, as well as familial history of OA, were registered. Familial history was defined as parents, siblings, or grandparents having a diagnosis of OA, having undergone arthroplasty of the knee or hip, or if they were reported to have Heberden’s nodes. Occupational risk, which was considered as occupational kneeling and lifting, and lifestyle habits such as smoking and drinking alcohol were registered. We also registered hormonal status in women; sport activities, defined as regular leisure activities; and the presence of previous knee injuries. Finally, we collected weight, height, and calculated BMI.

#### 2.3.2. Clinical Tests

##### Knee Morphology

Changes such as swelling, joint effusion, Baker’s cyst, or any others were documented.

##### Flexion Deformity

The patient was viewed from the side, and the long axis of the thigh and the leg were determined, and the angle between them was measured with the goniometer [13].

##### Leg Circumference

For leg circumference measurement, a manual circumference measurement at 10 cm from the upper side of the patella was used. A standard, non–elastic, bendable tape with a sensitivity level of 0.1 cm, using one-centimeter width for the measurements, was used. The tape was enclosed around the limb while the observer held the zero end of the tape with one hand and the other end of the tape with the other hand. Measurement results were observed from the point where the tape intersects with number zero and were recorded in centimeters to achieve standardization [14].

##### Knee Instability

To assess knee instability, the traditional passive tests were used. These tests included the Lachman test, the anterior/posterior drawer test, the pivot shift test, the quadriceps active test, and the varus/valgus stress test [15]. The primary structures that were tested were the anterior cruciate ligament, posterior cruciate ligament, and medial and lateral collateral ligaments [15].

##### Joint Proprioception

To measure knee joint proprioception, patient was in bedside-sitting position with legs out on the plinth and thighs fully supported. Subject was blindfolded to avoid any visual cues. Examiner passively extended knee joint from flexed position to the target angle of 30 degrees at very slow speed (about 10 degree/second). Subject attempted to identify test position whilst holding it actively for four seconds and then passively returned to the starting position. Then, subject was asked to reproduce target position actively using the same limb [16].

#### 2.3.3. Motor and Functional Variables

##### Knee Strength

For the specific evaluation of muscle strength, the isometric thigh muscle strength was used [17]. The evaluation was performed by a handheld dynamometer (HHD) as described by other authors [18]. The dynamometer used was the NedDFM/IBV employing the software NedDiscapacidad/IBV. Participants were asked to take a second or two to exert maximal effort and then continue trying to straighten their knee as vigorously as possible until the tester asked them to stop (about 4 s later). A total of 3 measures were performed and the average was used to quantify muscle strength [18].

**Figure 1:**
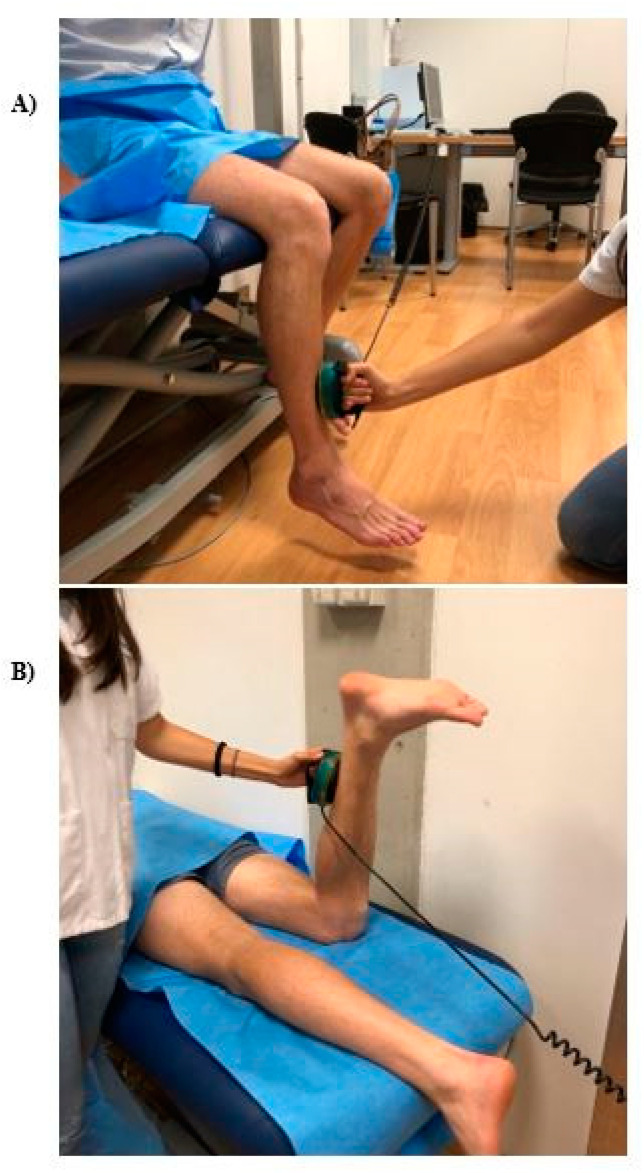
Knee strength measurement: (**A**) extension movement; (**B**) flexion movement.

To test the knee extension strength, subjects were seated, with their knees at about 90 degrees of flexion. HHD was placed against the anterior distal third of the leg of participants. To test the knee flexion strength, subjects were lying in prone position, with the knee under examination flexed about 90 degrees. HHD was placed against the posterior distal third of the leg of participants. Figure 1 represents the measurement protocol.

##### Knee Range of Motion

To obtain knees’ range of motion (ROM) measurements, particular care was taken to align the goniometer to the femur by palpating the greater trochanter and then aligning the proximal arm of the goniometer close to the femur. The distal arm of the goniometer was parallel to the tibia [19].

##### Sit-to-Stand

To assess functional capacity, the sit-to-stand test was employed. The test was performed as follows: with the patient seated with their back against the back of the chair, the clinician counted each standing movement aloud so that the patient remained oriented. The clinician stopped the test when the patient achieved the standing position on the 5th repetition [20].

##### Walking Speed

Subject walked without assistance 10 m (32.8 feet) and the time was measured for the intermediate 6 m (19.7 feet). Assistive devices could be used but had to be kept consistent and documented from test to test. Test was performed at the fastest speed possible. There were three trials collected and the average of the three trials was calculated for the measurement [21,22].

#### 2.3.4. Pain and Disability Variables

##### Pain Intensity

Visual Analogue Scale (VAS) was used to measure pain intensity. The VAS is a 100 mm line with two endpoints representing the extreme states “no pain” and “the maximal pain imaginable”. It has been shown to have good re-test reliability (r = 0.94, *p* < 0.001) and a minimal detectable change of 15.0 mm [23,24]. Pain intensity was measured both at rest and while walking.

##### Pain Type

Pain was also assessed with National Health and Nutrition Examination Survey (NHANES)-type pain questions, wherein duration of pain is indicated as ‘most days in a month’ (NHANES A and NHANES C), following published recommendations [25].

##### Western Ontario and McMaster Universities Osteoarthritis Index (WOMAC)

This instrument is the most extensively used for the functional and symptomatic assessment of patients with osteoarthritis. The WOMAC questionnaire is self-administered and is used to assess patients who progress with hip and/or knee osteoarthritis. The questionnaire is a multidimensional scale composed of 24 items divided into 3 aspects: functional pain (consisting of 5 items), stiffness (2 items), and activities of daily life difficulties (17 items). Higher values mean poorer WOMAC subscales scores of pain and physical function. The Spanish version of the WOMAC questionnaire has adequate psychometric properties, presenting an index of internal consistency (a) of 0.82 for pain and 0.93 for physical function subscales [26].

##### Knee Injury and Osteoarthritis Outcome Score (KOOS)

The KOOS is a knee-specific instrument, developed to assess a patient’s opinion about their knee and associated problems. The KOOS evaluates both short-term and long-term consequences of knee injury. It holds 42 items in 5 separately scored subscales: Pain, other Symptoms, Function in daily living (ADL), Function in Sport and Recreation (Sport/Rec), and knee-related Quality of Life (QOL). The psychometric properties and the ICC of the Spanish version have shown acceptable properties for both the total score and the subscales [27].

#### 2.3.5. Image Variables

##### Knee Alignment

Knee alignment was measured based on bilateral standard anterior–posterior weight-bearing radiographs as the angle formed by the intersection of the mechanical axes of the femur (the line from femoral head center to femoral intercondylar notch center) and the tibia (the line from ankle talus center to the center of the tibial spine tips). A knee was defined as varus when alignment was more than 0° in the varus direction, valgus when it was more than 0° in the valgus direction, and neutral when alignment was 0° (the angle made by the femur and tibia on a knee X-ray does not consider the proximal femur, femoral or tibial shafts, or ankle, so it is highly variable as opposed to full-limb measurements) [15].

##### Leg-Length Inequality (LLI)

We measured LLI in a bilateral standard anterior–posterior weight-bearing radiograph. We drew a line from the top of the femoral head that was lower than the other one. In addition, we then measured the distance between that line and the highest point of the other femoral head.

### 2.4. Procedures

An information sheet with an explanation of the procedure and an informed consent form were given to all the participants. Once the subjects had read the information from the study, they were allowed to ask any questions about its nature. The subjects that agreed to participate proceeded to fill in the sociodemographic questionnaire. Self-reported measures of disability, pain, and disability variables were then assessed. Finally, the physical examination was performed, including physical tests and motor and functional tests. The study protocol lasted approximately one hour. In order to avoid the influence of fatigue on the physical tests, an interval of 3 min between tests was maintained. This procedure was identical for both groups.

### 2.5. Statistical Analysis

The sociodemographic and clinical variables of the participants were analyzed. The data were summarized using frequency counts, descriptive statistics, summary tables, and figures. The data analysis was performed using the Statistics Package for the Social Sciences (SPSS 24, IBM Inc., Chicago, IL, USA). The categorical variables are shown as frequencies and percentages. The quantitative results are represented by descriptive statistics (confidence interval, mean, and standard deviation). For all variables, the z-score was assumed to follow a normal distribution based on the central limit theorem because all the groups had more than 30 participants [28,29]. Student’s *t*-test was used for group comparisons. Cohen’s *d* effect sizes were calculated for multiple comparisons of the outcome variables. According to Cohen’s method, the magnitude of the effect was classified as small (0.20–0.49), medium (0.50–0.79), or large (0.80).

The relationships between pain and disability measures and between physical measurements were examined using Pearson’s correlation coefficients. A Pearson’s correlation coefficient greater than 0.60 indicated a strong correlation, a coefficient between 0.30 and 0.60 indicated a moderate correlation, and a coefficient below 0.30 indicated a low or very low correlation [30].

## 3. Results

A total of 97 participants were included in the study, with a mean age of 51.51 ± 5.89 (36 men and 61 women). A total of 54 of the participants met the criteria to be classified as EOA while 43 were classified as HS. All the physical tests established in the protocol were performed on all the participants included in the study; no abandonment of any study participant was recorded. No adverse effects were reported during the assessments. There were no statistically significant differences between the groups in terms of the descriptive, demographic, and control variables (Table 1).

### 3.1. Clinical and Image Variables

Regarding differences in the clinical and image variables, the patients with EOA presented more knee pain (*p* < 0.01). In addition, more EOA patients showed instability both in the left (*p* < 0.01) and the right leg (*p* < 0.05). Significant differences were found with respect to the knee alignment variable (*p* < 0.04), wherein more patients with EOA diagnosed as possessing varus alignment. Detailed results are presented in Table 2.

### 3.2. Motor and Functional Variables

**Figure 2:**
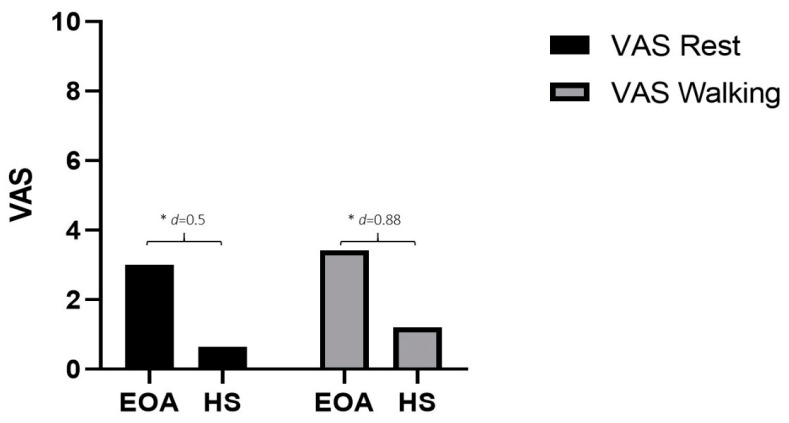
Differences in pain variable. * *p* < 0.05; *d*: Effect size; VAS: Visual Analogue Scale.

Regarding the differences between the groups, the patients with EOA showed higher levels of pain intensity at rest with a medium effect size (Mean Difference (MD): −2.35; *p* = 0.01; d = 0.5) and walking with a large effect size (MD: −2.21; *p* < 0.01; d = 0.88) (Figure 2).

**Figure 3:**
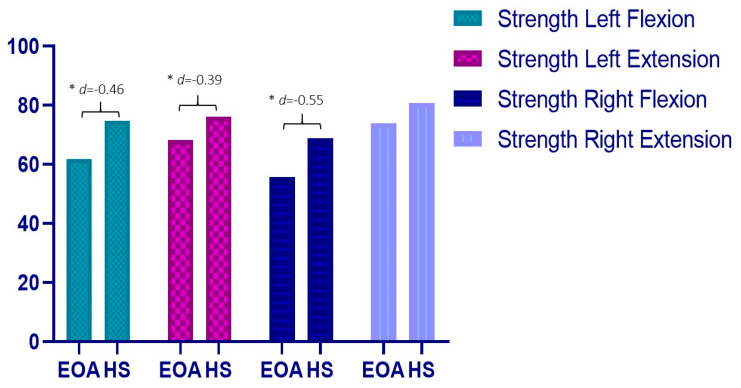
Differences in strength variable. * *p* < 0.05; *d*: Effect size.

In addition, the EOA patients showed lower knee strength, since statistically significant differences were found for flexion and extension strength in the left leg with a small effect size (MD: 12.92; *p* = 0.03; *d* = −0.46 and MD:7.81; *p* = 0.04; *d* = −0.39). In the right leg, similar significant differences were found between groups, but only regarding flexion movement, with a medium effect size (MD: 13.06; *p* = 0.01; *d* = 0.55) (Figure 3). No differences were found for the knee ROM values between groups.

Regarding the functional variables, statistically significant differences were found for the sit-to-stand test, showing more time expended for the EOA group, with a medium effect size (MD: −1.91; *p* < 0.01; *d* = 0.54) (Table 3).

### 3.3. Correlation Analysis

The strongest correlations were found in the EOA group between the left leg flexion strength, left knee ROM, and walking speed with walking VAS. These correlations were low (r = −0.33, r = −0.41 and r = 0.44; *p* < 0.05), and we also found the same trend for the right side, although it was not statistically significant. Correlations were also found between pain intensity at rest and ROM in both groups. Finally, correlations were found between the BMI and the ROM for left and right sides in the EOA patients (r = −0.66 and −0.55; *p* < 0.01) (Table 4).

## 4. Discussion

The main objective of this study was to evaluate which clinical, motor, or functional variables could be related to the appearance of EOA. Our study highlights some relevant data in relation to the early diagnosis of OA. In recent years, many efforts have been made to diagnose and classify these patients. However, this remains a challenge in such a heterogeneous disease, and our data provide new information about the risk factors and mechanisms involved in EOA [31]. Firstly, some of the risk factors (gender, personal history, or daily habits) previously identified in the scientific literature have not been found in our population with OAE. However, some other related physical or functional features have been associated with OA in this population, suggesting the importance of these factors in the diagnosis and prevention of OA. 

First, it is believed that non-modifiable factors such as age, gender, familial OA, and hormonal status could have an important role in the development of OA. Women have consistently been shown to be at higher risk of hip, knee, and hand OA, and whether this increase in risk is constant with age and changes in relation to endogenous estrogen production and menopause has been studied [32]. However, we found no differences in these variables. In addition, personal OA histories have been studied, with controversial results. Some studies have found the hand and/or hip OA history as one of the main factors that is consistently associated with knee OA [32], while other authors found it to be insignificant [8]. Our results follow the line of the latter authors, as we found no differences in the personal history of OA between subjects with EOA and HS.

On the other hand, some modifiable factors such as overweight and obesity (usually quantified using BMI), occupational risk, knee extensor muscle weakness, leg-length inequalities, or lifestyle habits could also significantly contribute to the onset of the pathology. A higher BMI was associated with greater knee pain accounting for OA severity in persons with or at a high risk for knee OA in the Osteoarthritis Initiative [33]. However, in our population, correlations were found between BMI and ROM in the subjects with EOA, but not with respect to pain intensity. Regarding lifestyle habits, drinking alcohol and smoking have been found to be statistically insignificant as either risk or protective factors [8,32]. Our data are in accordance with this statement, since no significant differences were found between the groups. The association of occupational activities with the development of knee OA has also been studied, finding an increased risk in floor-layers or in frequent occupational kneeling and lifting [34]. These differences were not found in our data, but it should be considered that our sample represented a small number of people with an occupational risk. Perhaps a larger sample of people with a high occupational risk would be necessary to detect differences in this aspect.

Regarding physical activity, a Cochrane Library’s review concluded that people are confused about the cause of their pain, and without adequate information from healthcare professionals, they avoid activity for fear of causing harm [35]. In addition, sport activities, defined as regular leisure activities, have been found to predict the progression of knee OA [34]. Being less sedentary was associated with better function in the Osteoarthritis Initiative, independent of moderate–vigorous physical activity minutes [36,37]. A greater degree of walking was associated with a lower risk of incident function limitation in persons with or at higher risk for knee OA in the Multicenter Osteoarthritis Study [38]. In our population, there seems to be a trend toward a greater sedentary lifestyle in the EOA group, but the differences were not significant.

The association of risk factors with the onset of knee pain has also been studied, establishing knee pain not as a predictor, but part of outcome measures for knee OA [8,25,32]. In our population, we combined radiographic and clinical criteria to classify patients into HS or EOA, as suggested by Luyten [11], and we found that the subjects with EOA showed higher levels of disability in terms of WOMAC and pain through VAS both at rest and during walking. In addition, our results show that higher levels of pain may be related to lower levels of ROM in both EOA and HS.

A systematic review and meta-analysis showed that knee extensor muscle weakness was associated with an increased risk of developing symptomatic knee OA [7]. Ruhdorfer et al., analyzing data from the Osteoarthritis Initiative, found that the reduction in thigh muscle strength in knee OA is related to pain but not to the structural (radiographic) disease status [39]. In this regard, our results showed that motor variables such as strength, or functional variables such as functional capacity, are significantly altered in patients with EOA compared to HS at risk of developing OA. In this regard, we also found differences between the right and left leg with respect to variables such as strength or ROM. The reasons for this may be varied, given that more subjects in both groups had pain or instability in the right leg due to a greater dominance of this leg with respect to the left.

Knee alignment, both static and dynamic, has important implications for load distribution within the knee. There is insufficient evidence to draw a conclusion regarding the effects of alignment on incident OA. It is possible that malalignment may be a reflection of the severity of the disease, with joint space loss due to cartilage and meniscal abnormalities, and bone contour alterations occurring as part of the OA disease process contributing to malalignment. In middle-aged, overweight women without knee OA in the Prevention of Knee Osteoarthritis in Overweight Females study, varus alignment was associated with incident radiographic OA; the association for valgus was borderline [40]. Similarly, LLI is an easily modifiable abnormality that can also affect lower extremity biomechanics. An LLI of at least 2 cm has been shown to be almost twice as likely to correspond to prevalent radiographic knee OA, but no such association was noted for incident knee OA [34]. Our data showed a predominance of varus alignment in the EOA group, suggesting its relevance in the development of EOA, but no significant differences were found with respect to LLI in our sample.

Previous studies suggest a relationship between instability and the onset and development of OA [41]. Our results are in line with these findings, as we found higher rates of subjects with ligamentous instability in the group of patients with EOA compared to healthy subjects. In this regard, it has also been suggested that previous knee injuries may contribute to the development of OA [8,34]. Although our results do not show significant differences between the groups, a higher percentage of injuries in the EOA group has been observed.

In addition, previous studies have shown that patients with higher levels of knee strength have higher levels of functional stability [42]. Our results show lower levels of strength in patients with EOA, suggesting that stability may also be dependent on this factor, and not just on ligamentous status. We hypothesize that these lower strength levels may lead to greater joint instability, which may play a role in the development of EOA, although future studies are needed to establish a cause–effect relationship. 

### Limitations

This research study has several limitations that should be considered when interpreting the results. First, the cross-sectional nature of this study makes it impossible to establish causality. In this sense, we did not find significant differences in many of the variables that have previously been identified as risk factors. This may be due to the fact that these variables (i.e., knee morphology, sports practice, or knee deformity) are not risk factors for the development of OA but rather consequences of the establishment of this pathology. This could entail that our sample would not present these alterations because we are dealing with an ‘early’ OA. Secondly, the lack of consensus and clear definition of EOA makes it difficult to draw clear conclusions about the differences between these groups of individuals. In this regard, several classifications have been proposed for EOA, and further research into the validity of these classifications is needed [43,44]. Third, in our sample, we have found a low percentage of patients presenting occupational risk, previous injuries, or toxic life habits, which have been previously related to the onset of OA. Studies with a larger sample size or in another type of population may be necessary to obtain relevant data in this regard. Finally, it would have been interesting to collect more information on physical activity in order to explore the implication of its intensity on the onset of EOA. 

## 5. Conclusions

The results of this research show statistically significant differences between patients with EOA and HS at risk of developing OA with respect to pain, disability, instability, knee strength, sit-to-stand test scores, and walking speed. Our results suggest that the evaluation of clinical, motor, and functional features could contribute to an early management of knee OA. However, further longitudinal research is needed to clarify the role of these variables in the onset and development of OA.

## Figures and Tables

**Table 1:** Descriptive, demographic data and control variables.

	EOA (*n* = 54)	HS (*n* = 43)	*p* Value
**Age (years)**	51.81 ± 5.65	51.05 ± 6.21	0.44
**BMI (kg/m^2^)**	27.28 ± 4.08	28.01 ± 2.96	0.59
**KL Grade**			0.67
0	19 (35)	23 (54)	
1	35 (65)	20 (46)	
**Gender**			0.78
Men	22 (40.8)	14 (32.5)	
Women	32 (59.2)	21 (67.5)	
**Hormonal status**			0.19
Pre-menopausal	12 (37.5)	14 (66.6)	
Post-menopausal	20 (62.5)	7 (33.3)	
**Smoking**			0.55
Yes	9 (16.6)	4 (9.3)	
No	21 (38.9)	19 (44.2)	
Ex	24 (44.5)	20 (46.5)	
**Alcohol**			0.66
Never	6 (11.1)	4 (9.3)	
Seldom	14 (25.9)	12 (27.9)	
1–2 times/month	13 (24.1)	11 (25.6)	
1–2 times/week	17 (31.5)	13 (30.3)	
1 time per day	3 (5.5)	2 (4.6)	
More than 1 a day	1 (1.9)	1 (2.3)	
**Previous Injuries Left**			0.35
No	42 (77.3)	27 (62.8)	
Yes	12 (22.2)	16 (37.2)	
Meniscus	8	3	
Ligament	0	4	
Bone	0	2	
Cartilage	1	2	
Unspecific	3	5	
**Previous Injuries Right**			0.47
No	40 (74.1)	25 (58.2)	
Yes	14 (25.9)	18 (41.8)	
Meniscus	4	7	
Ligament	5	2	
Bone	1	1	
Cartilage	1	2	
Unspecific	3	6	
**Knee morphology**			0.89
Normal	52 (96.3)	43 (100)	
Altered	2 (3.7)	0 (0)	
**Occupational risk**			0.68
Never	16 (29.6)	10 (23.3)	
Seldom	12 (22.2)	11 (25.6)	
1–2 times/month	3 (5.6)	5 (11.6)	
1–2 times/week	6 (11.1)	1 (2.3)	
1 a day	9 (16.7)	9 (20.9)	
Always	8 (14.8)	7 (16.3)	
**Family history**			0.56
Yes	38 (70.4)	32 (74.4)	
No	16 (29.6)	11 (25.6)	
**OA Hand history**			0.25
Yes	11 (20.4)	6 (14.0)	
No	43 (79.6)	37 (86.0)	
**OA Hip history**			0.66
Yes	8 (14.8)	7 (16.3)	
No	46 (85.2)	36 (83.7)	
**Sport**			0.54
Yes	24 (45.5)	27 (62.8)	
No	30 (55.5)	16 (37.2)	
**Education level** Primary Secondary College	9 (16.6) 19 (35.2) 26 (48.2)	6 (13.9) 15 (34.9) 22 (51.2)	0.92
**Marital status** Single Married Divorced Widow	6 (11.1) 36 (66.7) 10 (18.5) 2 (3.7)	7 (16.2) 32 (74.4) 2 (4.7) 2 (4.7)	0.22

The categorical variables are shown as frequencies and percentages. The quantitative results are represented by descriptive statistics (confidence interval, mean, and standard deviation). BMI: Body Mass index; EOA: Early Osteoarthritis; HS: Healthy subjects; KL: Kellgren-Laurence.

**Table 2:** Clinical and image variable results.

	EOA (*n* = 54)	HS (*n* = 43)	*p* Value
**Pain**			**<0.01**
**Yes**	**45 (83.3)**	**16 (37.2)**	
**No**	**9 (16.6)**	**27 (62.8)**	
**Pain side**			0.55
Left	7 (15.6)	3 (18.8)	
Right	14 (31.1)	4 (25)	
Both	24 (53.3)	9 (56.2)	
**NHANES Pain Left**			0.67
No pain	34 (63.0)	29 (67.5)	
A	10 (18.5)	9 (20.9)	
C	10 (18.5)	5 (11.6)	
**NHANES Pain Right**			0.2
No pain	30 (55.6)	29 (67.4)	
A	14 (25.9)	5 (11.6)	
C	10 (18.5)	9 (20.9)	
**Crepitus Left**			0.65
Yes	39 (72.2)	33 (76.7)	
No	15 (27.8)	10 (23.3)	
**Crepitus Right**			0.66
Yes	34 (62.9)	29 (67.4)	
No	20 (37.1)	14 (32.6)	
**Instability Left**			0.01
Yes	18 (33.3)	5 (11.6)	
No	36 (66.6)	38 (88.4)	
**Instability Right**			0.05
Yes	26 (48.1)	12 (27.9)	
No	28 (51.9)	31 (72.1)	
**Deformity Left**			0.69
Yes	16 (29.6)	13 (30.2)	
No	38 (70.4)	30 (69.8)	
**Deformity Right**			0.86
Yes	16 (29.6)	12 (27.9)	
No	38 (70.4)	31 (72.1)	
**Knee alignment Left**			0.04
Neutral	6 (11.1)	11 (25.6)	
Varus	32 (59.3)	16 (37.2)	
Valgus	16 (29.6)	16 (37.2)	
**Knee alignment Right**			0.47
Neutral	7 (12.9)	5 (11.6)	
Varus	32 (57.4)	21 (48.8)	
Valgus	15 (27.8)	17 (39.6)	
**Leg length inequality**			0.13
Yes	18 (33.3)	8 (18.6)	
No	36 (66.7)	35 (81.4)	
**Leg circumference**			0.32
Yes	42 (77.7)	34 (79.1)	
No	12 (22.3)	9 (20.9)	
**Proprioception Left**			0.88
Altered	44 (81.5)	32 (74.4)	
Normal	10 (18.5)	11 (25.6)	
**Proprioception Right**			0.33
Altered	35 (64.8)	31 (72.1)	
Normal	19 (35.2)	12 (27.9)	

The categorical variables are shown as frequencies and percentages. The quantitative results are represented by descriptive statistics (confidence interval, mean, and standard deviation). EOA: Early Osteoarthritis; HS: Healthy subjects.

**Table 3:** Between-group comparisons regarding pain, disability, motor, and functional variables.

Measures	EOA (*n* = 54)	HS (*n* = 43)	Mean Difference (95% CI)	Effect Size (*d*)
**VAS Rest**	3.0 ± 6.44	0.65 ± 1.53	−2.35 * (−4.35 to −0.35)	0.5
**VAS Walking**	3.42 ± 2.75	1.21 ± 2.23	−2.21 * (−3.24 to −1.18)	0.88
**WOMAC**	0.25 ± 0.13	0.12 ± 0.13	−0.13 ** (−0.19 to −0.7)	1.01
**KOOS**				
**Pain**	73.5 ± 13.79	89.29 ± 14.56	15.79 ** (10.01 to 21.56)	−1.11
**Symptoms**	77.87 ± 12.66	90.79 ± 12.76	12.91 ** (7.73 to 18.1)	−1.01
**ADL**	77.7 ± 15.94	90.83 ± 10.56	13.13 ** (7.47 to 18.79)	−0.97
**QQL**	52.63 ± 24.86	76.17 ± 23.96	23.54 ** (13.58 to 33.49)	−0.96
**Strength Left (Kg)**				
**Flexion**	61.91 ± 21.57	74.83 ± 33.00	12.92 * (0.45 to 25.39)	−0.46
**Extension**	68.24 ± 21.47	76.05 ± 17.81	7.81 * (1.26 to 16.88)	−0.39
**Strength Right (Kg)**				
**Flexion**	55.71 ± 16.52	68.77 ± 28.85	13.06 * (2.59 to 23.52)	−0.55
**Extension**	73.89 ± 22.98	80.86 ± 22.21	6.97 (−3.34 to 17.29)	−0.31
**ROM Left Leg (°)**	132.39 ± 9.61	132.43 ± 12.75	0.04 (−4.91 to 5.00)	−0.01
**ROM Right Leg (°)**	132.61 ± 8.11	131.84 ± 12.19	−0.78 (−5.29 to 3.74)	0.07
**Sit to stand (sec)**	13.18 ± 4.27	11.26 ± 2.61	−1.91 * (−3.52 to −0.31)	0.54
**Walking speed (sec)**	3.54 ± 0.89	3.36 ± 0.63	−0.17 (−0.53 to 0.18)	0.23

Note: ** *p* < 0.01; * *p* < 0.05; CI: confidence interval; EOA: Early osteoarthritis; HS: Healthy subjects.

**Table 4:** Correlation analysis.

Variable	Group	VAS Rest	VAS Walking	Strength Left F	Strength Left E	Strength Right F	Strength Right E	ROM Left	ROM Right	Sit-to-Stand	Walking Speed
***VAS Rest***	EOA	1	0.28 *	−0.01	0.15	−0.1	−0.05	−0.14	−0.32 *	0.02	0.04
	HS	1	0.69 **	−0.09	0.05	−0.16	−0.09	−0.62 **	−0.62 *	0.35*	0.4 *
***VAS Walking***	EOA	0.28 *	1	−0.33 *	−0.20	−0.13	−0.24	−0.41 **	−0.24	0.31 *	0.44 **
	HS	0.69 **	1	−0.13	−0.22	−0.22	−0.13	−0.28	−0.24	0.14	0.32
***BMI***	EOA	0.09	0.24	0.14	0.13	0.09	0.01	−0.66 **	−0.55 **	0.11	0.32 *
	HS	0.01	−0.03	0.27	0.28	0.25	−0.18	−0.31	−0.34	0.26	0.05
***WOMAC***	EOA	0.15	0.56 **	−0.12	−0.05	0.11	−0.15	−0.17	−0.27	0.27	0.19
	HS	0.52 **	0.63 **	−0.24	−0.17	−0.27	0.06	−0.08	−0.09	0.25	0.59 **

Note: ** *p* < 0.01; * *p* < 0.05; EOA: Early osteoarthritis; HS: Healthy subject.

## Data Availability

Not applicable.
